# BMP4 is increased in the aortas of diabetic ApoE knockout mice and enhances uptake of oxidized low density lipoprotein into peritoneal macrophages

**DOI:** 10.1186/1476-9255-10-32

**Published:** 2013-10-09

**Authors:** Mitsuhisa Koga, Atsushi Yamauchi, Yuki Kanaoka, Ryusuke Jige, Anna Tsukamoto, Nao Teshima, Tsuyoshi Nishioku, Yasufumi Kataoka

**Affiliations:** 1 Department of Pharmaceutical Care and Health Sciences, Faculty of Pharmaceutical Sciences, Fukuoka University, 8-19-1 Nanakuma, Jonan-ku, Fukuoka 814-0180, Japan

**Keywords:** BMP4, Atherosclerosis, Diabetes, Macrophage, Oxidized low density lipoproteins

## Abstract

**Background:**

BMP4, a member of the transforming growth factor-beta superfamily, is upregulated in the aortas of diabetic db/db mice. However, little is known about its role in diabetic atherosclerosis. Therefore, we examined the roles of BMP4 in the formation of diabetic atherosclerosis in apolipoprotein E knockout (ApoE KO) mice and in the uptake of oxidized low density lipoprotein (oxLDL) in peritoneal macrophages of wild-type mice.

**Methods:**

To induce diabetes, ApoE KO mice were intraperitoneally injected with streptozotocin. Diabetic and non-diabetic ApoE KO mice were then fed a high-fat diet for 4 weeks. Next, to investigate a role of BMP4 in the peritoneal macrophages, we examined the uptake of oxLDL in BMP4-treated macrophages.

**Results:**

Diabetic ApoE KO mice showed accelerated progression of aortic plaques accompanied by increased luminal plaque area. Western blot analysis showed that BMP4 expression in the whole aorta was greatly increased in diabetic ApoE KO mice, than non-diabetic mice. Western blot analysis showed that the BMP4/SMAD1/5/8 signaling pathway was strongly activated in the aorta from diabetic ApoE KO mice, compared with control ApoE KO mice. Double immunofluorescence staining showed that BMP4 was expressed in MOMA2-labeled macrophage in the aortic lesions of ApoE KO mice. BMP4 significantly increased the uptake of oxLDL into peritoneal macrophages in vitro.

**Conclusion:**

We show that in the aorta of diabetic ApoE KO mice, BMP4 is increased and activates SMAD1/5/8. Our in vitro findings indicate that BMP4 enhances oxLDL uptake in mouse peritoneal macrophages, suggesting BMP4 may be involved in aortic plaque formation in diabetic ApoE KO mice. Targeting BMP4 may offer a new strategy for inhibition of plaque progression and stabilization of atherosclerotic lesions.

## Introduction

Diabetes accelerates the progression of atherosclerosis, and induces vascular complications that are often life-threatening and disabling [1]. These complications represent a major clinical problem. There is increasing evidence that atherosclerosis is a chronic inflammatory disease in which inflammatory cells, including macrophages, monocytes, and T-lymphocytes, are recruited to and are activated in the atherosclerotic plaque by various cytokines and chemokines. Previous studies have revealed that oxidized low-density lipoprotein (oxLDL) is a causal factor for cardiovascular diseases [2-4]. An accumulation of oxLDL in foam cells derived from macrophages in atherosclerotic plaques causes plaque instability, before rupture [5,6]. These macrophages play key roles in all stages of atherosclerosis.

Bone morphogenetic proteins (BMPs) are bone-inducing morphogens and belong to the members of transforming growth factor-β superfamily [7,8]. BMPs also modulate cellular differentiation, proliferation, lineage determination, motility, and death [9-13]. Although the functions of BMPs in embryogenesis have been extensively studied, their roles after birth remain unclear [11].

In particular, BMP4 is expressed in calcified atherosclerotic plaques and aortic valve diseases [14], and these vascular BMPs contribute to the development of cardiovascular diseases. BMP4 is upregulated in *db/db* mice, an animal model of diabetes [15,16]. BMP4 was also reported to mediate monocyte adhesion, which is enhanced in atherosclerosis [17,18], restenosis [19], and diabetes [15].

Chronic BMP4 infusion causes endothelial dysfunction in a vascular NADPH oxidase-dependent manner in mice. Therefore, BMP4 is likely to be involved in the induction of hypertension [20]. Other BMPs, which have antagonistic properties, are coexpressed with BMP4 in mouse aortas and in human coronary arteries, suggesting that BMPs, including BMP4, are involved in the formation of atherosclerosis [21]. Taken together, these findings support the notion that BMPs play an important role in the pathophysiology of cardiovascular diseases and that they are key mediators of atherosclerosis. However, little is known about the role of BMP4 in macrophages in atherosclerotic plaques. Therefore, we examined the expression levels of BMP4 in atherosclerotic plaques of streptozotocin (STZ)-induced diabetic ApoE KO mice and the role of BMP4 in oxLDL uptake into macrophages in atherosclerotic lesions.

## Methods

### Animals

C57BL/6J ApoE KO mice were purchased from Jackson Laboratory (Bar Harbor, ME, USA) and were housed under standard conditions, including humidity, room temperature, and dark–light cycles. Mice were given free access to food and water throughout the study. The study protocol was approved by the Laboratory Animal Care and Use Committee of Fukuoka University. ApoE KO mice (6 weeks old) were intraperitoneally injected with 55 mg kg^–1^ day^–1^ STZ or vehicle (citrate buffer, control) over 5 consecutive days [22,23]. Blood glucose levels were measured 2 weeks after STZ administration to assess the induction of diabetes; only diabetic mice (defined as non-fasting glucose > 250 mg/dL) were used in this study. Both groups of ApoE KO mice were fed a high-fat diet (1.25% cholesterol, 15% cacao butter, and 0.5% sodium cholate, F2HFD1, Oriental Yeast CO, Tokyo, Japan) for 4 weeks, starting from 8 weeks old. At 12 weeks old, the animals were killed, and the aortas removed for comparisons between STZ-induced diabetes and control mice.

### En-face plaque area

To quantify the extent of atherosclerotic lesions, immediately after the mice were killed, the whole length of the aorta (n = 9 mice in each group) was excised for quantification of the *en face* plaque area, as previously described [24,25]. Briefly, after carefully removing adventitial tissue, the aortic arch and the thoracic to abdominal aorta were opened longitudinally, pinned on a black wax surface, and stained with Oil red O (Sigma, St. Louis, MO, USA). *En face* images were obtained by a stereomicroscope and analyzed using a public domain software Image J (NIH Image, Bethesda, MD, USA) (n = 9 mice/group). The percentage of the luminal surface area stained by Oil red-O was determined [24,25].

### Histology

After the mouse was sacrificed and perfused with ice-cold phosphate-buffered saline (PBS), the heart and the ascending aorta were removed *en bloc* and snap-freezed in O.C.T. compound (Sakura FineTech, Tokyo, Japan) for histological and immunohistochemical analyses. Serial cryostat sections (6 μm thick) of the aortic root were prepared as previously described [24,25]. Briefly, atherosclerotic plaques were examined in five independent sets of sections taken 60 μm apart. Oil red O staining was performed to identify the lipid-rich core. The Oil red O-stained areas, as a marker of lipid accumulation, were analyzed using Image J software. In each mouse, the mean for five independent sections was used for the analysis.

### Double immunofluorescence staining

For staining the frozen sections, fresh mouse aortas were excised from ApoE KO mice, placed in Tissue-Tek O.C.T. compound, snap-freezed in lipid nitrogen, and stored at -80°C until use. After removing the O.C.T. compound and blocking, the samples were incubated with antibodies against BMP4 (1:100; Santa Cruz Biotechnology Inc., Santa Cruz, CA, USA) and MOMA2 (1:500; BMA Biomedicals, Augst, Switzerland) overnight at 4°C. For double-immunofluorescence staining, the samples were incubated with FITC (Nacalai Tesque, Kyoto, Japan) and AlexaFluor 594®-conjugated secondary antibodies (Molecular Probes, Eugene, OR, USA), respectively, for 1 h at room temperature. Nuclei were counterstained with DAPI (Vector Laboratories, Burlingame, CA, USA). MOMA2-stained areas, as a marker of macrophage accumulation, were analyzed using Image J software.

### Western blotting

The aorta was immediately snap-freezed in liquid nitrogen. Aortic proteins were isolated using lysis buffer (50 mM HEPES, 50 mM NaCl, 5 mM EDTA, 1% Triton X-100, 1 mM Na_3_VO_4_, 50 mM NaF, 10 mM sodium pyrophosphate decahydrate and 1 mM phenylmethylsulfonyl fluoride), containing 1% phosphatase inhibitor cocktail 1 (Sigma), 1% phosphatase inhibitor cocktail 2 (Sigma), and 1% protease inhibitor cocktail (Sigma). After tissue homogenization, particulate material was removed by centrifugation, and protein concentrations measured using the Bio-Rad protein assay (Bio-Rad Laboratories, Hercules, CA, USA). Equal amounts of total protein (30 μg/sample) were subjected to sodium dodecyl sulfate–polyacrylamide gel electrophoresis (10%) and electrophoretically transferred to polyvinylidene difluoride membranes (Millipore, Billerica, MA, USA). Non-specific antibody binding was blocked by incubating the membranes with Blocking One (Nacalai Tesque) for 60 min at room temperature. The primary antibodies used were mouse monoclonal anti-BMP4 (1:100, Santa Cruz Biotechnology Inc.) and anti-SMAD1 (1:100, Santa Cruz Biotechnology Inc.), and rabbit polyclonal anti-phospho-SMAD1/5/8 (1:1000, Cell Signaling, Danvers, MA) and anti-β-actin (1:2000, Abcam, Cambridge, UK). Blots were incubated overnight at 4°C with primary antibodies, and then incubated with horseradish peroxidase-conjugated secondary antibodies (1:5000 dilution) for 60 min at room temperature. The blot was developed using an ECL detection kit (Amersham International, Little Chalfont, UK). Signal intensities were normalized using beta-actin. Band images were digitally captured with a FluorChem SP imaging system (Alpha Innotech, San Leandro, CA, USA) and band intensities quantified using Image J software.

### Preparation of peritoneal macrophages

To isolate peritoneal macrophages, we intraperitoneally injected wild-type mice with 2 mL of 4% thioglycollate. Cells were collected from the peritoneal cavity 3 days after injection and were incubated in 12-well plates in complete medium (RPMI1640 media containing 10% fetal bovine serum and 100 U/mL penicillin/streptomycin). After 2 h, the cells were washed three times with PBS and cultured in media. The adherent cells, considered to be peritoneal macrophages, were used in the experiments [26]. For a single experiment, peritoneal macrophages were collected from one mouse.

### Uptake of oxLDL into peritoneal macrophages

**Figure 1:**
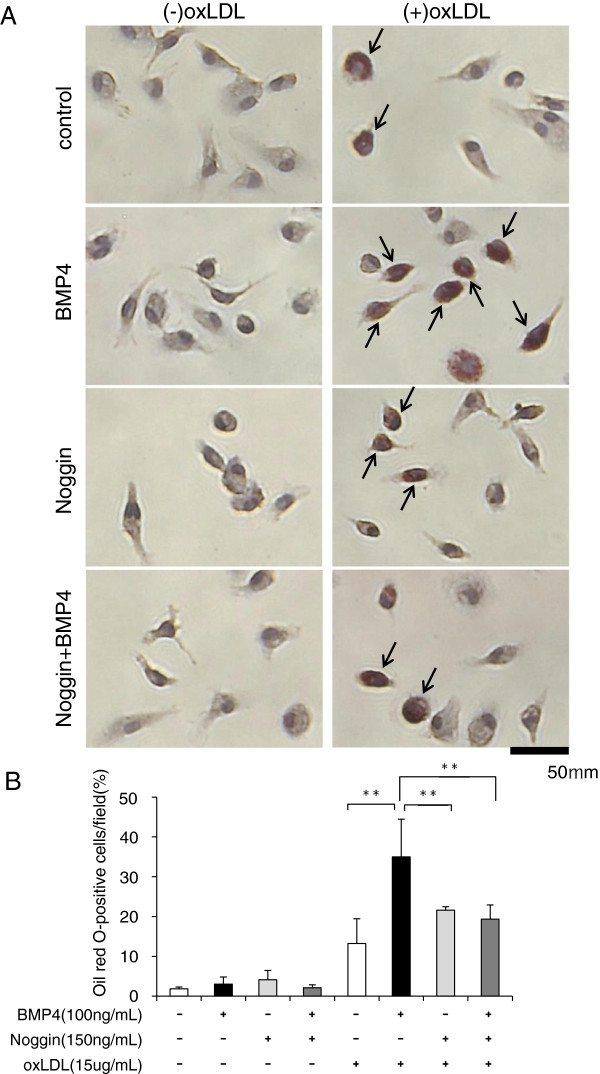
**Lipid accumulation determined by Oil red-O staining in peritoneal macrophages.** To inhibit BMP4, peritoneal macrophages were incubated with Noggin (150 ng/ml) for 3h. After pretreatment of Noggin, cells were incubated with or without BMP4 (100 ng/ml) in the presence or absence of oxLDL (15 μg/ml) for 20 h. **(A)** Arrow heads show Oil red-O-positive cells in the representative images. **(B)** Quantitative analysis of oxLDL uptake as a percentage of Oil red-O-labeled cells in total cells. Each bar indicates mean ± S.D. of four separate experiments, with each experiment performed in triplicate.

OxLDL was purchased from HARBOR BIO-PRODUCTS (Norwood, MA, USA). Recombinant BMP4 and Noggin (a BMP4 antagonist) were purchased from R&D systems (Minneapolis, MN, USA). Peritoneal macrophages were serum starved for 3 h and then pretreated with or without 150 ng/ml Noggin for 3 h. OxLDL (15 μg/ml) and BMP4 (100ng/ml) were added to Noggin-treated or untreated peritoneal macrophages for 20 h. After washing three times with PBS, cells were fixed in 4% paraformaldehyde, and stained with Oil red-O and hematoxylin to evaluate uptake of oxLDL. The average percentage of Oil red O-labeled macrophages for each well was obtained by counting the number of labeled and unlabeled cells in 15 microscopic fields selected from each of three separate wells/group. The average value was calculated for each group. The experiment was repeated four times and the average percentage of four separate experiments was shown in Figure 1C.

### Measurement of blood glucose and plasma cholesterol levels

At 12 weeks of age, blood was collected to measure blood glucose, plasma total cholesterol and triglyceride levels. Glucose was measured directly from the tail tip with a glucometer (Glutest sensor, Sanwa Kagaku Kenkyusho, Mie, Japan). Plasma total cholesterol and triglyceride levels were determined using commercial available kits (Wako Chemical Co., Osaka, Japan).

### Statistical analysis

All quantitative analyses were performed by a single observer blinded to the experimental protocol. Data are expressed as means ± standard deviation. Differences among the two groups were compared using unpaired Student’s *t*-test. The statistic comparison among the 8 groups was performed using ANOVA followed by Tukey’s Multiple Comparison tests. Values of P < 0.01 were considered to be statistically significant.

## Results

### Plasma lipid and lipoprotein levels in diabetic ApoE KO mice

ApoE KO mice were rendered diabetic with an intraperitoneal administration of STZ, and were then fed a high-fat diet for 4 weeks. Approximately 80% of the mice were diabetic at 2 weeks after STZ administration, defined as non-fasting glucose > 250 mg/dL. Six weeks after STZ, glucose levels in the control and diabetic ApoE KO mice were 102 ± 12 and 454 ± 123 mg/dL, respectively (Table 1). After being fed the high-fat diet for 4 weeks, fasting plasma cholesterol levels in the diabetic ApoE KO mice were 1.4-fold higher than those in the control mice (2529 ± 513 vs. 1815 ± 302 mg/dL). Plasma triglyceride levels were not significantly different between the two groups of mice (n = 18, control mice; n = 16, diabetic ApoE KO mice).

**Table 1:** Non-fasting glucose, lipid, and lipoprotein levels in control and diabetic mice

	**cont**	**STZ**
Body Weight (g)	23.6 +/- 2.8	21.2 +/- 3.5*
Glucose (mg/dl)	102 +/- 12	454 +/- 123**
Total Cholesterol (mg/dl)	1815 +/- 302	2529 +/- 513**
Triglycerides (mg/dl)	499 +/- 74	548 +/- 164

Data are expressed as mean +/- S.D. obtained from 16 diabetic ApoE mice and 18 control mice. *P<0.05 and **P<0.01 vs control mice.

### Atherosclerotic plaques in the aorta

**Figure 2:**
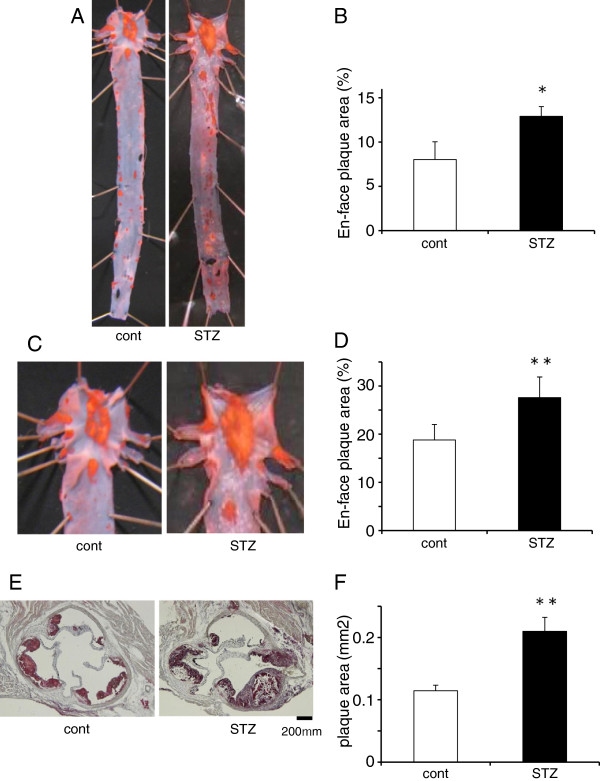
**Atherosclerotic plaques in the aorta. (A**, **C**, **E)** Representative photomicrographs of control (cont) and diabetic ApoE KO mice (STZ) showing that STZ-induced diabetes accelerates the progression of atherosclerotic lesions in the whole aorta, aortic arch, and aortic root. **(B**, **D**, **F)** Quantitative measurement of the *en face* plaque area (%) in the whole aorta **(B)** and aortic arch **(D)**, and oil red-O-stained plaque area (mm^2^) in the aortic root **(F)** (n = 9 mice/group). Each bar indicates mean ± S.D. *P < 0.05 and **P < 0.01 vs control mice.

At 12 weeks of age, the control and diabetic ApoE KO mice were killed to measure plaque area. The non-diabetic control ApoE KO mice had Oil red O-stained atherosclerotic plaques extending from the ascending aorta to the aortic arch. By contrast, the diabetic mice had more severe aortic plaque formation, with an increase in *en face* plaque area of 15% (Figure 2A–D). In addition, STZ-induced diabetes markedly accelerated the formation of atherosclerotic plaques in the aortic sinus (Figure 2E,F).

### BMP4 protein expression in the aorta

**Figure 3:**
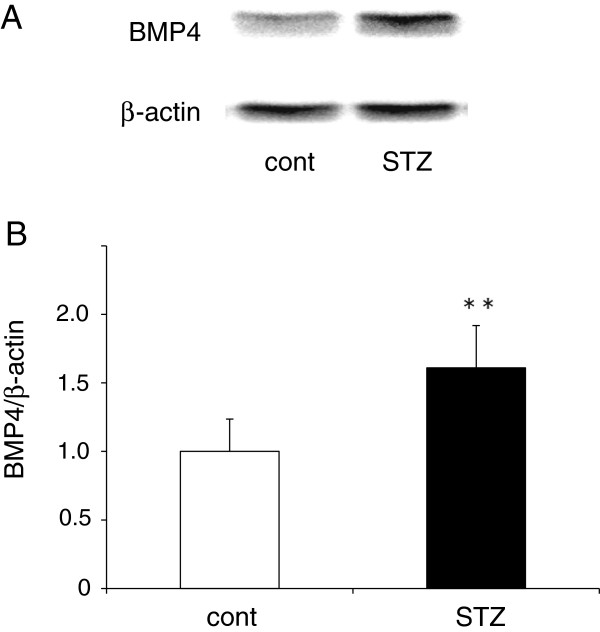
**BMP4 protein expression in the aorta. (A)** Representative western blots showing BMP4 protein expression in the whole aortas from control (cont) and diabetic ApoE KO mice (STZ). **(B)** Scanning densitometry quantitative analysis of BMP4 protein expression (BMP4 protein/β-actin; n = 9 mice/group). Data are expressed as a ratio relative to the control group. Each bar indicates mean ± S.D.* *P < 0.01 vs control mice.

BMP4 protein expression levels in the aortas of control and diabetic ApoE KO mice were evaluated by western blot. Aortic BMP4 protein expression was significantly increased, by approximately 60%, in diabetic ApoE KO mice compared with control ApoE KO mice (n = 9 mice/group) (Figure 3B). Cross-sections taken at the aortic sinus were evaluated to determine the structural features of the atherosclerotic lesions. At 12 weeks of age, the plaques in the aortic root consisted of a lipid-rich core and massive macrophage infiltration into the intima in control ApoE KO mice. By contrast, the formation of lipid- and macrophage-rich plaques was remarkably increased at the aortic root in the diabetic ApoE KO mice.

**Figure 4:**
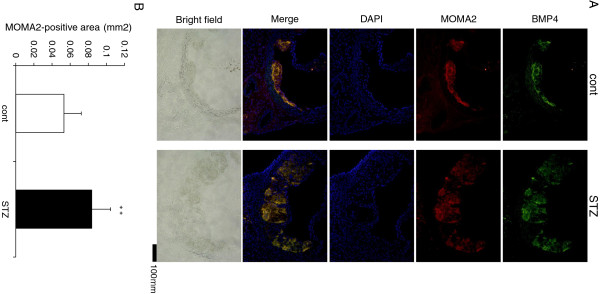
**Expression of BMP4 in monocytes/macrophages in the aortic roots of control (cont) and diabetic ApoE KO mice (STZ). (A)** Representative images showing the localization of BMP4 (green), MOMA2 (red) and DAPI (blue) staining in each group. Frozen sections of the aortic roots were stained with anti-BMP4 and anti-MOMA2 antibodies, and were then observed under a fluorescence microscope. **(B)** In each group, the MOMA2-positive area (mm^2^) in the aortic root was measured in five separate sections from 8 mice. Results are presented as the average area. Each bar indicates mean ± S.D. **P < 0.01 vs control mice.

Next, to test whether BMP4 was expressed in atherosclerotic lesions in the aortic sinus of ApoE KO mice, we stained several sections of the aortic root with anti-BMP4 antibody. The low magnification images (Figure 4A) revealed BMP4 protein in the internal layer. The localization of BMP4 in the atherosclerotic lesion was confirmed by double-immunofluorescence staining with anti-MOMA2 antibody (specific for monocytes/macrophages) and anti-BMP4 antibody. The BMP4-positive area appeared to correspond to the macrophage-rich area of the atherosclerotic lesion in the aortic root (Figure 4A). Furthermore, MOMA2-stained areas were larger at the aortic root in diabetic ApoE KO mice, than in controls (Figure 4B). BMP4 expression in monocytes/macrophages in the atherosclerotic lesions was much greater in diabetic mice than in control ApoE KO mice.

### Phosphorylation of SMAD1/5/8 signaling in the aorta

**Figure 5:**
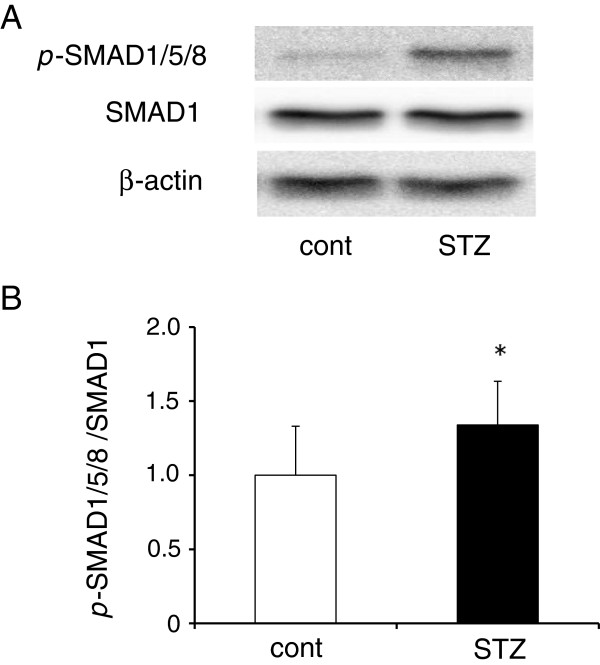
**The BMP4 signaling pathway is activated in atherosclerotic lesions in STZ group.** Representative western blots **(A)** and densitometric analysis **(B)** of *p*-SMAD1/5/8 and SMAD1 in each group (n = 6 mice/group). A ratio of *p*-SMAD1/5/8 to SMAD1 was calculated from band intensities and data expressed as a ratio relative to the control group (n = 6 mice/group). Each bar indicates mean ± S.D. *P < 0.05 vs control mice.

Western blot analysis showed SMAD1/5/8 phosphorylation was clearly induced in the whole aorta in diabetic ApoE KO mice, compared with controls (Figure 5A). Summarized data of SMAD1/5/8 phosphorylation is shown (Figure 5B; n = 6/group).

### BMP4 increases oxLDL uptake in the peritoneal macrophages

OxLDL incorporation in peritoneal macrophages obtained from wild-type mice was markedly increased by BMP4 treatment compared with untreated peritoneal macrophages (Figure 1). Noggin, a BMP4 antagonist, inhibited BMP4-induced oxLDL uptake in peritoneal macrophages. In the absence of oxLDL, few Oil red-O-positive peritoneal macrophages were observed in each group. On the other hand, we observed few Oil red-O-positive peritoneal macrophages in the absence of oxLDL. BMP4 alone did not increase the number of Oil red-O positive peritoneal macrophages (Figure 1).

## Discussion

Diabetes leads to the progression of atherosclerotic lesions, coronary artery disease, stroke, and peripheral vascular disease [27-29]. Atherosclerosis, an inflammatory disease, is thought to occur as a result of the uptake of oxLDL into macrophages/monocytes [30-33]. Current clinical strategies have focused on lipid lowering with statins, for example, to prevent the progression of atherosclerosis.

The present study provided the first experimental evidence to show that BMP4 enhances oxLDL uptake into peritoneal macrophages. We also found that BMP4 protein expression was markedly upregulated in the aorta of STZ-induced diabetic ApoE KO mice, compared with controls (Figure 3). Recent findings suggest that BMP4 may function as a pro-inflammatory and pro-atherogenic vasculature mediator [15]. We showed that BMP4 protein expression was elevated (Figure 4A) in parallel with increased accumulation of MOMA2-stained macrophages (Figure 4B) in atherosclerotic plaques from diabetic ApoE KO mice. These findings suggest that increased BMP4 expression in aortic macrophages of diabetic ApoE KO mice, may be involved in enhanced oxLDL uptake. In the present study, we induced diabetes in ApoE KO atherosclerotic mice by injecting them with STZ [22,23]. These mice developed marked hyperglycemia, with blood glucose levels > 250 mg/dL. STZ also increased the plasma total cholesterol levels in the ApoE KO mice but did not affect triglyceride levels compared with the control ApoE KO mice (Table 1).

As shown in Figure 2, atherosclerotic plaque formation was accelerated in the whole aorta, aortic arch, and aortic root of diabetic ApoE KO mice. These observations indicate that diabetes accelerates atherosclerotic plaque formation. BMP4 expression was also much greater in the whole aortas of diabetic ApoE KO mice compared with control mice (Figure 3), suggesting that diabetes also induces aortic BMP4 expression in *db/db* mice [15]. BMP4 induces the activation of the SMAD1/5/8 signaling pathway. In this study, diabetic ApoE KO mice showed strong activation of BMP4/SMAD1/5/8 signaling in aortas compared with control ApoE KO mice due to increased expression of BMP4 in the diabetic aortas (Figures 3, 4, and 5). These data suggest that BMP4 may be one of the important regulators to progress plaque formation underlying diabetes diseases. There is evidence indicating that BMP antagonists and signaling pathway inhibitors block activation of SMAD1/5/8 signaling, and thereby reduce the incidence of subsequent events, including vascular inflammation and atherosclerosis [34,35]. These findings suggest that BMP signals are novel therapeutic targets for vascular inflammation and/or atherosclerosis.

To examine the localization of BMP4 expression in the aorta, we performed double-fluorescence staining of monocytes/macrophages and BMP4. The BMP4- and monocyte/macrophage-positive areas were largely colocalized in the atherosclerotic plaque of aortic roots, as shown in Figure 4A. Lesional monocytes and macrophages are the main cell types involved in the progression of atherosclerotic plaques, because the phagocytic activity of macrophages in the plaque contributes to the development of atherosclerosis and plaque instability. BMP4 treatment increased 2.6-fold the number of cells with oxLDL uptake, when compared with controls (12% of the total) (Figure 1). This marked increase in macrophages showing oxLDL uptake was significantly inhibited by 50% when cells were treated with Noggin. These results suggest that the increase in BMP4 expression associated with diabetes will enhance the uptake of oxLDL into macrophages in atherosclerotic lesions. Therefore, it is very likely that diabetes accelerates the formation of atherosclerotic plaques and lowers the threshold for destabilization and rupture of atherosclerotic lesions.

In conclusion, we have demonstrated that BMP4 is expressed in monocytes/macrophages in atherosclerotic plaques in a mouse model of diabetes and atherosclerosis. We also found that BMP4 enhances oxLDL uptake into peritoneal macrophages in vitro. The induction of BMP4 in atherosclerotic plaque may promote atherosclerotic plaque formation in diabetes. These findings raise the possibility that inhibition of BMP4 signaling may represent a potential therapeutic target for atherosclerosis and other diseases associated with BMPs and diabetes.

## Competing interests

The authors declare that they have no competing interests.

## Authors’ contributions

MK: Conceived, designed and conducted experiments for the study and wrote the manuscript. YK, RJ, AT, NT: Performed experiments and helped in constructing the figures. TN: Participated in the study design and coordination and drafting of manuscript. AY: Contributed to supervision of laboratory procedures, data analysis and interpretation. YK: Conceived and designed the study, contributed to data analysis and interpretation and wrote the manuscript. All authors have read and approved this manuscript.
